# Effects of various doses of lubabegron on calculated ammonia gas emissions, growth performance, and carcass characteristics of beef cattle during the last 56 days of the feeding period

**DOI:** 10.1093/tas/txab137

**Published:** 2021-08-21

**Authors:** John C Kube, Ben P Holland, Alyssa B Word, Janet B Allen, Michelle Calvo-Lorenzo, David McKenna, Gary Vogel

**Affiliations:** 1 Elanco, Greenfield, IN 46140, USA; 2 Cactus Research, Amarillo, TX 79116, USA; 3 Tyson Fresh Meats Inc., Dakota Dunes, SD 57049, USA

**Keywords:** ammonia gas emissions, agonist/antagonist, beta adrenergic modulator, cattle carcass characteristics, lubabegron, mobility

## Abstract

Lubabegron (LUB; Experior, Elanco, Greenfield, IN, USA) was approved by the U.S. Food and Drug Administration in 2018 and is indicated for the reduction of ammonia (NH_3_) gas emissions·kg^−1^ body weight (BW) and hot carcass weight (HCW) when fed to feedlot cattle during the final 14 to 91 d of the finishing period. LUB demonstrates antagonistic behavior at the β _1_ and β _2_ receptor subtypes and agonistic behavior at the β _3_ receptor subtype in cattle and is classified by the Center for Veterinary Medicine as a “beta-adrenergic agonist/antagonist.” This report describes a randomized complete block study that evaluated LUB dose (0, 1.5, 3.5, and 5.5 mg·kg^−1^ dry matter) during the last 56 d of the feeding period on calculated NH_3_ gas emissions, live weight, carcass weight, and associated ratios in beef feedlot cattle. Carcass characteristics, mobility, and health were also evaluated. All cattle received monensin and tylosin throughout the study. Ammonia gas emissions were calculated using the equation developed by Brown et al. (Brown, M. S., N. A. Cole, S. Gruber, J. Kube, and J. S. Teeter. 2019. Modeling and prediction accuracy of ammonia gas emissions from feedlot cattle. App. Anim. Sci. 35:347–356). The reduction in calculated cumulative NH_3_ gas emissions with LUB ranged from 1.3% to 11.0% (85 to 708 g/hd). When NH_3_ gas emissions were expressed on a live weight (unshrunk) and carcass weight basis, calculated NH_3_ gas emissions decreased by 3.0% to 12.8% and 3.8% to 14.6%, respectively. Daily dry matter intake was 2.3% greater (*P*_trt_ < 0.05) for steers that received LUB. Average daily gain was 13.7% greater (*P*_trt_ < 0.05; 1.68 vs. 1.91 kg), while gain efficiency was 10.8% greater (*P*_trt_ < 0.05; 0.167 vs. 0.185) for steers fed LUB. Animal mobility was scored in the pen approximately 1 wk prior to harvest, when cattle were loaded on trucks scheduled for harvest, and at antemortem inspection during lairage. No treatment differences (*P*_trt_ ≥ 0.170) were observed at any time for the percent of cattle receiving mobility scores of 1 or 2 (normal or minor stiffness but moving with the normal cattle, respectively). Cattle mobility scored as a 1 or 2 equaled or exceeded 92% at all times. Final BW and HCW increased (*P*_trt_ < 0.05) 11.6 to 15.7 kg and 11.3 to 17.1 kg, respectively, in cattle receiving LUB compared to cattle receiving monensin plus tylosin alone.

## INTRODUCTION

Emissions from animal feeding operations may contribute to regional, national, and global air-shed pollution (NRC, 2003), with ammonia (NH_3_) gas implicated in ecosystem nutrient enrichment, reduced visibility, and diminished air quality through the formation of fine particulate matter (PM_2.5_) (NOAA, 2014). In 2008, the Environmental Protection Agency (EPA) exempted confined animal feeding operations from reporting NH_3_ gas emissions under the Comprehensive Environmental Response, Compensation, and Liability Act (CERCLA), but required feedlots with daily NH_3_ gas emissions above 45 kg or permitted capacities greater than 1,000 animals to report under the Emergency Planning and Community Right-To-Know Act (EPCRA; Waldrip et al., 2015). A ruling by United States Court of Appeals for the District of the Columbia Circuit (Waterkeeper Alliance v. U.S. EPA, 2017) removed the reporting exemptions in CERCLA and EPCRA for all confined animal feeding operations. Subsequently in 2019, the EPA amended the release notification regulations under the EPCRA to add the reporting exemption for air emissions from animal waste at farms (Federal Register, 2019). Although not regulated, actions and measures to reduce NH_3_ gas emissions demonstrate beef producers’ commitment to environmental sustainability and improvement.

In 2018, lubabegron (LUB; Experior, Elanco, Greenfield, IN, USA) was approved for reducing NH_3_ gas emissions·kg^−1^ final body weight (BW) and hot carcass weight (HCW) when administered to cattle during the last 14 to 91 d on feed. The approval marked the first time the U.S. Food and Drug Administration (FDA) approved a drug that reduces gas emissions from an animal or its waste. Clinical effectiveness studies demonstrated a reduction in NH_3_ gas emissions·kg^−-1^ BW and HCW when LUB is fed during the last 14 to 91 d of the finishing period (FDA-FOI, 2018). The objective of this study was to provide information for feeding LUB at 0, 1.5, 3.5, or 5.5 mg·kg^−1^, 100% dry matter (DM) basis to finishing cattle for the last 56 d of the finishing period.

The pharmacological category assigned by the Center for Veterinary Medicine (CVM) for LUB is β-adrenergic agonist/antagonist (FDA-FOI, 2018). Because LUB binds to β receptors (FDA-FOI, 2018), it is defined as a β ligand. The metabolic activity of LUB in a given tissue, such as skeletal muscle, depends on the density and subtype of the β receptor, that is β _1_, β _2_, or β _3_.

LUB is unique in that it is an antagonist at the β _1_ and β _2_ receptors (FDA-FOI, 2018), meaning it blocks stimulation of those receptor subtypes. Conversely, LUB is an agonist at the β _3_ receptor (FDA-FOI, 2018), meaning that it stimulates that receptor subtype. Because of its dual modulating, blocking, and activating effect, LUB is most appropriately classified as a β modulator. Additionally, LUB selectively binds to β adrenergic receptors (binding affinity observed at ≤ 0.5 nM) and has low binding affinity for non-β adrenergic receptors (i.e., no affinity observed at > 300 nM for muscarinic, 5-HT_2_, dopamine D_1_ and D_2_, α _1_- and α _2_-adrenergic, benzodiazepine, histamine H_1_, or GABA_A_ receptors) (FDA-FOI, 2018). Because of its selectivity and modulating characteristics, LUB can simply and accurately be described as a selective β modulator.

## MATERIALS AND METHODS

This study was conducted at Cactus Research, Amarillo, TX, USA. Animal care and disposal methods were in accordance with applicable Federal, State, and Local regulations. All study procedures were reviewed and approved by Elanco’s Institutional Animal Care and Use Committee (IACUC; Approval number EIAC-0827).

### Experimental Design and Treatments

A randomized complete block design was used to evaluate the effects of LUB on calculated NH_3_ gas emissions and growth performance of 2,880 British and Continental European crossbred steers typical for U.S. feedlots. *Bos indicus* breeding was limited to less than 1/8. The study evaluated four LUB doses: 0 (control, CON), 1.5, 3.5, and 5.5 mg·kg^−1^ DM. There were 12 blocks, each block containing four replicate pens that started LUB feeding on the same date. Steers were randomized within each block of four pens with 60 steers per pen, resulting in a total of 720 steers enrolled in each treatment. LUB feeding started on June 24, 2018, July 01, 2018, and July 15, 2018, four blocks on each date. The ratio of calculated cumulative NH_3_ gas emissions (CCAGE) to final BW and HCW (kg) was calculated over a 56-d period. The study was powered based on a denominator variable (i.e., HCW) since the numerator was a calculated value and the denominator was based on actual animal or carcass weight. The study was powered to detect a 5.4 kg HCW response at 80% power between CON and individual treatment doses.

### Study Timeline and Treatment Allocation

On or prior to d -85 (i.e., 85 days prior to slaughter) and before randomization, animals eligible for enrollment were sorted based on BW, days on feed, phenotype, health, and general disposition, with the most uniform approximately 1,000 steers selected for potential enrollment in each time replicate group. Animals were examined by a qualified evaluator and screened for eligibility based on the following inclusion criteria: confirmed to be a steer and determined to be in good health. Steers received a Revalor XS implant (trenbolone acetate and estradiol; Merck Animal Health, Summit, NJ) 4.7 to 7.0 months before slaughter (i.e., d 0). Animals with existing or pre-existing abnormal health conditions or observations were eligible for inclusion in the study if the condition was considered to be minor in nature (e.g., minor lacerations, eye redness, dermatitis, etc.) and not expected to worsen or impact the animal’s ability to grow normally and complete the study. Animals that appeared unthrifty, ill, or injured were excluded. In addition, animals were excluded due to extreme BW. Randomization of animals to pens occurred 66 to 69 d before slaughter, which was considered the start of the approximately 10 to 15 d acclimation phase. The mean BW of the 12 blocks ranged from 538.4 to 570.6 kg. The minimum and maximum within-block BW range across all 12 blocks were 106.1 and 118.8 kg, respectively. These data suggest the cattle were uniform across blocks and within each block to ensure acceptable uniformity of cattle at harvest.

Cattle were assigned into one of four blocks during each randomization event (i.e., time replicate, *n* = 3). For each time replicate, there were four blocks of four pens (one pen for each dose level).

Allotment of animals to pens was conducted 9 to 12 d prior to the first feeding of experimental diets. The experimental diets were fed for 56 d. Complete blocks (four pens, one pen per treatment group) were allocated on a given day. Pens contained 60 steers each and were contiguous in proximity. The randomization schedule was prepared using a Microsoft Excel spreadsheet. At the time of randomization, steers were individually weighed and assigned to study pens if within target weight range of ± 56.7 kg. Each succeeding group of four candidate steers was placed into one of four pens within the block using the randomization schedule. Subsequent blocks were populated by repeating the same procedure. A separate randomization schedule was used to assign one of the four treatments to each of the four pens in each block.

### Ammonia Gas Emissions Calculations

The following equation was used to determine cumulative NH_3_ gas emissions on the study (Brown et al., 2019):


$$\begin{array}{l} \begin{array}{lll} & y\left(log_{10}\left[cumulative\;NH_{3}\;gas\;emissions,\;g\cdot animal^{-1}\right]\right)= & \\ & \;0.06758372\;\times \left(cumulative\;N\;intake,\;kg\cdot animal^{-1}\right)\\ & -0.011425\;\times \left(lubabegron\;dose,\;g\cdot ton^{-1}\right)\\ & -4.9743281\;\times \left(1\cdot cumulative\;duration^{-1},\;d\right)\\ & -0.0012361{\;\times \left(cumulative\;N\;intake,\;kg\cdot animal^{-1}\right)}^{2}\\ & +0.0002744{\;\times \left(outdoor\;temperature,\;{}^{\circ }C\right)}^{2}+3.01996229 \end{array} \end{array}$$

where:

LUB dose is on a 100% DM basis.

Cumulative N intake per animal = sum of weekly average feed consumed adjusted for the number of animals in each pen each day × (assayed crude protein [CP; %] ÷ 6.25) from weekly feed samples collected during the treatment period.

Outdoor temperature = average of the daily mean ambient temperature during the treatment period. Daily ambient temperature was obtained from Hale County airport (KPVW) in Plainview, TX.

CCAGE were used to determine CCAGE·final BW^−1^ (g·kg^−1^) and CCAGE·HCW^−1^ (g·kg^−1^), the primary outcomes of interest.

### Health Observations

During the approximately 10 to 15 d acclimation phase and the entire treatment phase until cattle were loaded for slaughter, all animals were observed by trained caretakers at least once daily, but health issues were only noted by exception. All abnormalities were recorded even if considered common for feedlot cattle. Abnormal health observations were observations that the observer considered as 1) typical for beef cattle at that age, or 2) not causing undue pain or distress to the animal, and/or, 3) not impeding the animal’s growth at the time the observation was made. Animals with abnormal health observations were allowed to remain in the study. Animals were allowed to receive concomitant therapies (e.g., antibiotic treatment for respiratory disease and treatment for bloat).

Health observations requiring an animal to be classified as “removed” were abnormalities that the observer considered as 1) resulting in pain or distress to the animal, or 2) likely to result in further deterioration of the animal’s health, and/or 3) impairing the animal’s ability to access food or water at the time of the observation. Animals removed from the study during the treatment or withdrawal phase were penned separately in the research feedlot and tracked through slaughter to assure accountability of all cattle on study per food use authorization or were euthanized and necropsied. Decisions to remove an animal from treatment phase were made by the Investigator or Manager.

Animals that were found dead at the time of the observation were removed from the pen as soon as practical and necropsied (if possible).

### Diet Formulation and Feed Assays

Beginning no later than approximately d -70, animals were fed a diet containing Rumensin (monensin 46.3 mg·kg^−1^ 100% DM basis; Elanco, Greenfield, IN, USA) and Tylan (tylosin 8.9 mg·kg^−1^ 100% DM basis; Elanco) ad libitum. Cattle in the LUB treatments were switched from the basal finishing ration to the finishing ration containing the appropriate concentrations of LUB Type A premix at the start of the treatment period (d -57). Table 1 lists the composition and the formulated nutrient composition of the finishing diet. The diet was designed to meet or exceed the minimum nutrient requirements cited in Nutrient Requirements of Beef Cattle (NASEM, 2016). LUB was delivered to the cattle using a 1% LUB Type A premix prepared and delivered by Elanco (Clinton Laboratories, Clinton, IN, USA), and the ration was prepared on-site at the research facility. The 1% Type A premix was delivered into the ration by flushing the 1% Type A premix through a micro-ingredient machine (Micro Technologies, Amarillo, TX) with water, which was included at approximately 27 kg of the “as-fed” ration, for each 3,629 kg batch. The 1% Type A premix was included in the finishing ration at 0, 0.0154%, 0.0353%, or 0.0551% (100% DM) to provide CON, 1.5, 3.5, or 5.5 mg·kg^-1^ LUB (100% DM).

**Table 1.** Ingredient composition, analyzed nutrient content, and the formulated nutrient and monensin/tylosin composition of the finishing diet fed during the acclimation and treatment phases

Ingredient composition, % dry matter (DM)^1^	
Flaked corn	57.3
Wet distillers grains	17.3
Sweet bran plus^2^	17.0
Cotton burrs	7.1
Corn oil	1.3
Water plus micro-ingredients^3^^,^^4^	Approximately 0.05
Total	100.0
Formulated nutrient and monensin/tylosin concentration	
DM	63.1
Crude protein (CP), % of DM	14.6
Degradable intake protein, % of DM	7.8
Crude protein equivalent from non-protein nitrogen (NPN), % of DM	0.8
Ether extract, % of DM	5.7
Neutral detergent fiber (NDF), % of DM	19.9
Physically effective NDF, % of DM	11.2
Ca, % of DM	0.8
P, % of DM	0.5
K, % of DM	0.7
Mg, % of DM	0.2
Salt, % of DM	0.5
S, % of DM	0.2
Co, mg·kg^−1^ of DM	0.5
Cu, mg·kg^−1^ of DM	13
I, mg·kg^−1^ of DM	0.5
Fe, mg·kg^−1^ of DM	84
Mn, mg·kg^−1^ of DM	45
Se, mg·kg^−1^ of DM	0.36
Zn, mg·kg^−1^ of DM	79
Calculated NE_m_^5^, Mcal·kg^−1^ of DM	2.15
Calculated NE_g_^5^, Mcal·kg^−1^ of DM	1.49
Calculated monensin^6^, mg·kg^−1^ of DM	46.3
Calculated tylosin^7^, mg·kg^−1^ of DM	8.9

^1^The mean of wet chemistry analysis (DM basis of diet) of 11 weekly ration samples collected during the treatment phase of the study for each of the four treatment groups was 15.5% CP, 0.86% Ca, 0.48% P, 1.2% NPN, 2.0% acid-detergent insoluble nitrogen (ADIN). The range of mean CP for the four treatment groups was 15.4% to 15.7%. Samples analyzed by Servi-Tech Laboratories (Amarillo, TX).

^2^Sweet Bran wet corn gluten feed (Cargill Corn Milling, Bovina, TX) with added (as fed) calcium carbonate (4%), salt (1.8%), urea (1.1%), and trace mineral premix (0.2%).

^3^Water was included at 27.2 kg per batch through the micro-ingredient machine for inclusion of lubabegron, monensin, and tylosin. Each batch of manufactured feed weighed approximately 3,629 kg.

^4^Formulated to provide the following in each kilogram of total diet DM: 2,645 IU Vitamin A, 265 IU Vitamin D, 46.3 mg monensin, 8.9 mg tylosin, and CON, 1.5, 3.5, or 5.5 mg of lubabegron.

^5^Calculated based on NASEM (2016).

^6^Provided at approximately 464 mg·hd^−1^·day^−1^.

^7^Provided at approximately 90 mg·hd^−1^·day^−1^.

The on-site feed mixers used to mix (stationary horizontal paddle mixer [Cactus Varied Industries, Amarillo, TX, USA]) and deliver (Roto-Mix 490-14 and Roto-Mix 620-16, Roto-Mix LLC, Dodge City, KS) the Type C feed were qualified to confirm that the ration could be mixed homogeneously before feeding medicated feed. Cattle were fed in order of CON, 1.5, 3.5, or 5.5 mg·kg^−1^ doses of LUB. Prior to mixing the CON batches, the mixer was flushed with appropriate amount of non-medicated feed or other cattle feed to assure no carryover of test compound. Feed was issued three times each day at approximately the same time each day. Bunk calls were made at the time of first feeding and amount of feed issued for the third feeding was adjusted based on daily bunk calls according to site procedures. The feeding goal was to have slick bunks prior to the first feeding and approximately 1/3 of the cattle at the bunk, 1/3 of the cattle moving to the bunk, and 1/3 of the cattle not moving to the bunk at time of first feeding. Water was available ad libitum throughout the study. The weight of feed issued was recorded and electronic feed records were provided for data analysis.

Samples (separate samples for LUB and nutrient analysis) were collected weekly from each treatment of ration prepared (d -57 to d -1) for analysis. Duplicates consisting of a composite of three samples from different locations within the delivered truck load were used for either a primary or a back-up sample. The weekly primary samples were analyzed for LUB at Eurofins Laboratories (Greenfield, IN). Back-up samples were frozen. Feed assays were performed using a validated analytical method for LUB (Determination of Lubabegron in Medicated Feed by High-Performance Liquid Chromatography: Laboratory Procedure G1635). The permissible analytical variation of LUB content in a single feed analysis (weekly composite) was ± 25% for the 1.5 mg·kg^−1^ truck-loads and ± 20% for the 3.5 and 5.5 mg·kg^−1^ truck-loads.

Samples for nutrient analysis were submitted fresh on the day the samples were collected to Servi-Tech Laboratories, Inc. (Amarillo, TX). Analysis included DM (National Forage Testing Association procedure #2.1.4 Dry Matter by Oven Drying for 3 hr at 105 °C), CP (AOAC #990.03), N (% CP ÷ 6.25), non-protein nitrogen (NPN) (AOAC #941.04), acid detergent insoluble nitrogen (ADIN) (AOAC #2001.11), and Ca and P (AOAC #990.08).

### Feeding and Growth Performance

Animals were weighed individually at the time of randomization (approximately d -69 to d -66) and this weight was used to exclude steers with extreme BW from the trial. All other BW (unshrunk) were collected by pen and used for calculation of growth performance. Scheduled pen weights were taken prior to feed delivery and collected on d -59 (2 d before treatment start, which was considered the treatment start weight) and d 0 (1 d after the end of treatment, which was considered the treatment end weight). Prior to each collection of animal weights, the scales were qualified following site operating procedures.

Feed weighback (if feed remained in bunk) occurred on d -57, d -1, d 0, and whenever required due to soiled, spoiled or excessively wet feed. When feed weighbacks occurred between d -59 and d 0, a composite sample of weighback from all bunks was collected and DM determined on each sample on-site.

Average daily DMI was calculated by subtracting weighbacks from the amount offered the previous day as-fed and then multiplying intake as-fed by the dietary DM and adjusting for total animal-days during the treatment period. Average daily gain (ADG) (kg·hd^-1^) was calculated for the entire 56 d treatment period using unshrunk BW (kg) excluding the animals removed during the treatment period. Gain efficiency was summarized as the gain:feed quotient (ADG:DMI).

### Animal Mobility

Animal mobility assessments were conducted three times prior to harvest at: 1) approximately 1 wk prior to harvest while cattle were in their home pens; 2) after collection of final pen weights and immediately prior to loading into trucks for slaughter; and 3) at antemortem inspection during lairage at the packing plant. The mobility scorer used the North American Meat Institute’s (NAMI) mobility scoring system (Edwards-Callaway et al., 2017) to evaluate cattle mobility. The NAMI mobility scoring system is a 4-point scoring system where 1 = normal, walks easily with no apparent lameness or change in gait; 2 = keeps up with normal cattle when the group is walking, exhibits one or more of the following: stiffness, shortened stride, or slight limp; 3 = lags behind normal cattle when the group is walking, exhibits one or more of the following: obvious stiffness, difficulty taking steps, obvious limp, or discomfort; 4 = extremely reluctant to move, even when encouraged by handlers.

### Slaughter and Carcass Characteristics

LUB was removed from feed at least 24 h before harvest to comply with the Food Use Authorization (FDA, 2016) granted by the FDA. Cattle were loaded for harvest shortly after collection of final pen live weights and transported to the slaughter facility at a stocking density of approximately 30 steers (i.e., half a pen of 60 steers) in one semi-trailer. The slaughter process was conducted in accordance with USDA requirements and standard slaughter site procedures. Animal/carcass identification was maintained by sequentially recording the ear tag numbers of cattle as they were slaughtered via a sequence number affixed to each carcass by trained personnel from the Beef Carcass Research Center (BCRC; West Texas A&M University, Canyon, TX) and correlating the individual ear tag number to the plant-assigned carcass ID number, which remained intact throughout the carcass data/sample collection phase. Following an industry-standard dressing procedure, each carcass was weighed for determination of HCW.

Carcass evaluation occurred following the commercial plant’s standard chilling period. The carcass quality and yield grade factors, marbling score, ribeye area, and objective 12th rib fat thickness were captured objectively using the VBG2000 camera system. Additionally, adjusted 12th rib fat thickness, kidney, pelvic, and heart (KPH) fat assessment, and other defects (dark cutters, blood splash, etc.) were recorded by trained personnel (BCRC). Skeletal maturity was obtained only on carcasses that were “B” maturity or greater, otherwise, skeletal, lean, and overall maturity and lean color were not obtained. Carcass defects (dark cutters, excessive trim) were noted by exception. The objective fat thickness was used to calculate the USDA yield grade. All carcasses were considered to be less than 30 mo of age; therefore, USDA quality grade was determined by using marbling score (USDA, 2019). The severity of the dark cutting condition was indicated by assigning the dark cutting carcasses to 1/3, 2/3, or full dark categories. Dark cutters were not excluded when determining quality grade, however, a summary of the frequency of dark cutter carcasses by treatment group is provided. Carcasses identified as excessively trimmed (greater than approximately 9.1 kg trim) were not excluded from the analysis, however, a summary of the frequency of excessively trimmed carcasses by treatment group is provided.

### Statistical Analysis

The label claim variable of cumulative NH_3_ gas emissions over the treatment period normalized by final BW (with d -59 BW as a covariate) for the period and HCW (g of gas·kg^−1^ of BW or g of gas·kg^−1^ HCW) was determined by utilizing NH_3_ gas emissions equations developed from the LUB clinical effectiveness study (FDA-FOI, 2018) and validated using animals of similar BW as described by Brown et al. (2019).

The pen was the experimental unit for each outcome. Differences were deemed significant using a two-sided test at *P*_trt_ ≤ 0.05, however, a less stringent significance threshold of *P*_trt_ ≤ 0.10 was used for abnormal health observations. Fixed effect of treatment and random effects of time replicate and block within time replicate were included in the model.

Discrete variables were analyzed using a generalized linear mixed model, Proc GLIMMIX SAS version 9.4 unless there were convergence problems due to sparseness of data. A binomial distribution was assumed and a logit link used in the analysis. Contrasts were constructed between the CON group and each non-CON dosage group, and differences were deemed significant at *P*_dose_ ≤ 0.05. If convergence problems arose due to sparseness of data, Fisher’s exact test (binomial data; Proc FREQ in SAS) or Wilcoxon’s rank-sum test (categorical data; Proc NAR1WAY in SAS) was used to evaluate differences between the CON group and the non-CON dosage groups. In all cases except for health observations, differences were deemed significant at *P*_trt_ ≤ 0.05 for these primary discrete variables. Additionally, non-CON dosage groups were compared to one another using a significance level of *P*_dose_ ≤ 0.05. A less stringent *P*_trt_ ≤ 0.10 level of significance was used for health observations.

Continuous variables were analyzed using a linear mixed model, Proc MIXED SAS version 9.4. Differences were deemed significant at *P*_trt_ ≤ 0.05 for the discrete variables. As required by protocol, d -59 BW was included as a covariate in the analysis of the primary non-claim variables (ANCOVA): final BW, HCW, and DMI. This covariate remained in the model regardless of its statistical significance. Contrasts were constructed between the CON group and each non-CON group, and differences were deemed significant at *P*_dose_ ≤ 0.05. The variables with a statistically significant contrast were tested to determine if dose response followed a linear or quadratic fit. Additionally, non-CON groups were compared to one another using a significance level of *P*_dose_ ≤ 0.05.

## RESULTS

### Feed Composition and LUB Assays

The ingredient composition, analyzed nutrient content and the formulated nutrient and monensin/tylosin composition of the finishing diet fed during the 56 d treatment phase are presented in Table 1. The LUB assay results (mean of the 11 weekly samples collected during the treatment phase and range) for the CON, 1.5, 3.5, or 5.5 mg·kg^−1^ treatment groups were 1.34 (1.19 to 1.47), 3.13 (2.89 to 3.56), and 4.93 (4.63 to 5.28) mg·kg^−-1^, respectively, on a 100% DM basis. All LUB feed assay results were within the specified acceptable range. Daily LUB consumption was 0, 13.8, 32.6, and 50.8 mg·hd^−1^ for the CON, 1.5, 3.5, or 5.5 mg·kg^−1^ treatment groups, respectively.

### Animal Health

Table 2 summarizes the abnormal health observations and animal removals that occurred after treatment initiation. No significant (*P*_trt_ > 0.10) between-treatment differences were observed. Total removals (animals found dead and all animals removed from the treatment phase for health reasons) for each treatment group were 1.7, 2.5, 1.5, and 1.8% for CON, 1.5, 3.5, or 5.5 mg·kg^−1^ treatment groups, respectively. All cattle passed routine USDA antemortem inspections. Two steers were not harvested due to hyperactive disposition. One steer (3.5 mg·kg^−1^ treatment group) was euthanized at the packing plant and not harvested. The second steer (CON group) was returned to the research facility and slaughtered at a later date. Carcass data from these two steers were not included in the analysis.

**Table 2.** Abnormal health observations after treatment initiation presented as total number and rate (%)^1^

Clinical system	Clinical sign	Lubabegron^2^ dose, mg·kg^−1^ DM				
		Control	1.5	3.5	5.5	P_trt_^3^
Musculoskeletal	Lameness	7 (1.0)	9 (1.3)	3 (0.4)	7 (1.0)	0.444
Gastrointestinal	Bloat^4^	0	1 (0.1)	4 (0.6)	2 (0.3)	≥0.124
Respiratory	Pneumonia	3 (0.4)	4 (0.6)	6 (0.8)	2 (0.3)	0.539
Removals^5^		12 (1.7)	18 (2.5)	11 (1.5)	13 (1.8)	0.547
General	Dead^6^	1 (0.1)	2 (0.3)	5 (0.7)	2 (0.3)	0.379

^1^60 hd per pen; 12 pens per treatment; 720 total hd per treatment. Rate (%) of abnormal health was calculated based on 720 hd per treatment.

^2^Experior, Elanco, Greenfield, IN, USA.

^3^Significant effect: *P*_trt_ ≤ 0.10.

^4^Health observations did not converge, therefore, Fisher’s Exact Test for each contrast was performed where animal was the experimental unit. The total number of animals per dose group was 720 hd.

^5^Removals include animals found dead (i.e., System: General; Sign: Dead) and all animals removed from study during the treatment phase.

^6^Animals found dead.

### Animal Mobility

Table 3 summarizes the animal mobility assessments that were conducted prior to harvest. No differences (*P*_trt_ ≥ 0.17) among treatments for the percent of cattle scored 1 were observed at any time. Cattle scoring a 1 or 2 equaled or exceeded 92% of the animals at all times. Animals receiving abnormal scores of >2 were not different across treatments at any of the assessment time points prior to harvest.

**Table 3.** Animal mobility scores prior to and at harvest in steers fed lubabegron^1^ for the final 56 d of the feeding period

Scoring location^2^	Mobility score^3^	Lubabegron dose, mg·kg^−1^ DM Percentage of steers				P_trt_^4^
		Control	1.5	3.5	5.5	
Pen	1	86.3	85.0	85.9	86.9	0.793
	2	9.9	11.4	10.4	8.3	0.299
	>2	3.8	3.6	3.7	4.8	0.619
Loadout	1	74.6	76.6	73.7	71.3	0.170
	2	23.9	21.4	23.1	25.0	0.440
	>2	1.5	2.0	3.2	3.7	0.062
Lairage	1	82.7	80.2	81.7	80.9	0.644
	2	10.9	11.8	12.1	12.6	0.786
	>2	6.4	8.0	6.2	6.5	0.523

^1^Experior, Elanco, Greenfield, IN, USA.

^2^Animal mobility assessments were conducted at: Approximately 1 wk prior to harvest while cattle were in their home pens (“Pen”), after collection of final pen weights and immediately prior to loading into trucks for slaughter (“Loadout”) and during antemortem inspection during lairage at the packing plant (“Lairage”).

^3^Cattle mobility was scored by trained observer using a 4-point scale from the North American Meat Institute (NAMI, 2015).

Score 1 = Normal, walks easily, no apparent lameness, no change in gait.

Score 2 = Exhibits minor stiffness, shortness of stride, slight limp, keeps up with normal cattle.

Score 3 = Exhibits obvious stiffness, difficulty taking steps, obvious limp, obvious discomfort, lags behind normal cattle.

Score 4 = Extremely reluctant to move even when encouraged by a handler, statue-like.

^4^Significant effect: *P*_trt_ ≤ 0.05.

### Cumulative Ammonia Gas Emissions

Cattle treated with LUB experienced a 1.3% to 11.0% (85 to 708 g) reduction in CCAGE when compared to CON cattle (overall treatment effect for each is *P*_trt_ < 0.001; Table 4). In addition, there was a 3.0% to 12.8% reduction in CCAGE when standardized by BW, and a 3.8% to 14.6% reduction in CCAGE when standardized by HCW versus CON cattle (overall treatment effect for each is *P*_trt_ < 0.001; Table 4). Compared to CON and to each dose group of LUB, the reductions in CCAGE standardized by BW and HCW were greater (*P*_dose_ < 0.05) as the LUB dose increased.

**Table 4.** Least squares means for the effects of lubabegron^1^ (LUB) dose on calculated cumulative NH_3_ gas emissions (CCAGE) and CCAGE standardized by final body weight (BW) and hot carcass weight (HCW) over 56 d

	LUB (mg·kg^−1^ DM)								Difference from control^2^		
Variable	Control	1.5	3.5	5.5	SEM	P_trt_^3^	P_Lin_^4^	P_Quad_^5^	1.5 mg·kg^−1^ vs. control	3.5 mg·kg^−1^ vs. control	5.5 mg·kg^−1^ vs. control
Final BW^6^^,^^7^, kg	667.4	679.0	683.1	681.7	2.7	<0.001	<0.001	<0.001	11.6 (1.7) 3^,x^	15.7 (2.4) 3^,y^	14.3 (2.1) 3^,x,y^
HCW^6^, kg	408.0	419.3	423.9	425.1	1.4	<0.001	<0.001	<0.001	11.3 (2.8) 3^,x^	15.9 (3.9) 3^,y^	17.1 (4.2) 3^,y^
NH_3_ gas emissions											
CCAGE^6^, g· animal^−1^	6460	6375	6075	5752	120	<0.001	<0.001	0.047	−85 (−1.3)^x^	−385 (−6.0)^3^^,y^	−708 (−11.0)^3^^,z^
Standardized by BW BW^6^^,^^7^, g·kg^−1^	9.68	9.39	8.89	8.44	0.15	<0.001	<0.001	0.641	−0.29 (−3.0)^3^^,x^	−0.79 (−8.2)^3^^,y^	−1.24 (−12.8)^3^^,z^
Standardized by HCW^6^, g·kg^−1^	15.8	15.2	14.3	13.5	0.25	<0.001	<0.001	0.876	−0.6 (−3.8) 3^,x^	−1.5 (−9.5) 3^,y^	−2.3 (−14.6)^3^^,z^

^1^Experior, Elanco, Greenfield, IN, USA.

^2^Control values subtracted from treatment values. Values in parenthesis are percent change from Control.

^3^Different from Control *P* ≤ 0.05.

^4^Significance for linear contrast.

^5^Significance for quadratic contrast.

^6^Initial animal weight used as a covariate.

^7^Based on unshrunk BW.

^x,y,z^Least squares means with different superscripts differ *P* ≤ 0.05.

### Growth Performance and Carcass Characteristics

Initial BW was not different (*P*_trt_ = 0.709) for cattle assigned to different treatments. However, final BW and HCW increased (*P*_trt_ < 0.001) 11.6 to 15.7 kg and 11.3 to 17.1 kg, respectively, in cattle receiving LUB compared to CON (Table 5). Average daily gain increased (*P*_trt_ < 0.001) 11.3% to 15.5% and DMI increased (*P*_trt_ = 0.025) 2.0% to 3.0% in LUB-fed steers compared to CON. Gain efficiency (ADG: DMI) improved (*P*_trt_ < 0.001) 9.0% to 12.0% for cattle treated with LUB compared to CON.

**Table 5.** Least squares means for the effects of lubabegron^1^ (LUB) on growth performance traits and carcass characteristics of beef cattle over a 56 d treatment period

	LUB (mg·kg^−1^ DM)								Difference from control^2^		
Variable	CON	1.5	3.5	5.5	SEM	P_trt_^3^	P_Lin_^4^	P_Quad_^5^	1.5 mg·kg^−1^ vs. control	3.5 mg·kg^−1^ vs. control	5.5 mg·kg^−1^ vs. control
Growth performance											
Initial body weight (BW)^6^, kg	567.7	569.0	569.5	567.6	2.3	0.709	Not Determined	Not Determined	1.3	1.8	−0.1
Final BW^6^^,^^7^, kg	667.4	679.0	683.1	681.7	2.7	<0.001	<0.001	<0.001	11.6 (1.7)^3^^,x^	15.7 (2.4)^3^^,y^	14.3 (2.1)^3^^,x,y^
Average daily dry matter intake (DMI)^7^, kg·hd^−1^	10.1	10.3	10.4	10.3	0.25	0.025	0.013	0.060	0.2 (2.0)^3^	0.3 (3.0)^3^	0.2 (2.0)^3^
Average daily gain (ADG)^6^, kg·hd^−1^	1.68	1.87	1.94	1.92	0.05	<0.001	<0.001	<0.001	0.19 (11.3)^3^	0.26 (15.5)^3^	0.24 (14.3)^3^
Gain:feed, ADG:DMI^6^	0.167	0.182	0.187	0.186	0.003	<0.001	<0.001	<0.001	0.015 (9.0)^3^	0.020 (12.0)^3^	0.019 (11.4)^3^
Carcass characteristics											
Hot carcass weight (HCW)^7^, kg	408.0	419.3	423.9	425.1	1.4	<0.001	<0.001	<0.001	11.3 (2.8)^3^^,x^	15.9 (3.9)^3^^,y^	17.1 (4.2)^3^^,y^
Dressing percentage^6^	61.1	61.8	62.1	62.3	0.11	<0.001	<0.001	0.014	0.7^3^^,x^	1.0^3^^,y^	1.2^3^^,z^
Adjusted fat thickness, cm	1.54	1.53	1.48	1.48	0.06	0.038	<0.007	0.761	-0.01^x^	−0.06^3^^,x,y^	−0.06^3^^,y^
Ribeye area, cm^2^	93.7	97.0	99.1	99.4	1.7	<0.001	<0.001	<0.001	3.3 (3.5) 3^,x^	5.4 (5.8) 3^,y^	5.7 (6.1) 3^,y^
Marbling score^8^	473	460	450	446	4.6	<0.001	<0.001	0.097	−13.0 (−2.8)^3^^,x^	−23.0 (−4.9)^3^^,y^	−27.0 (−5.7)^3^^,y^
Yield grade^9^	3.21	3.14	3.02	3.01	0.15	<0.001	<0.001	0.193	−0.07 ^x^	−0.19^3^^,y^	−0.20^3^^,y^
KPH fat, %	2.11	2.12	2.12	2.14	0.03	0.774	Not Determined	Not Determined	0.01	0.01	0.03

^1^Experior, Elanco, Greenfield, IN, USA.

^2^Control values subtracted from treatment values. Values in parenthesis are percent change from Control.

^3^Different from Control *P* ≤ 0.05.

^4^Significance for linear contrast.

^5^Significance for quadratic contrast.

^6^Growth performance and dressing percentage were based on unshrunk initial and final BW.

^7^Initial animal weight used as a covariate.

^8^400 = Small^0^ (low choice); 500 = Modest^0^ (middle choice); 600 = Moderate^0^ (high choice). Determined by objective measurement with a camera.

^9^Yield Grade = 2.50 + (0.98 × adj. fat thickness, cm) + (0.2 × KPH fat, %) + (0.0038 × HCW, kg) – (0.32 × ribeye area, cm^2^).

^x,y,z^Least squares means with different superscripts differ *P* ≤ 0.05.

**Figure 1.**
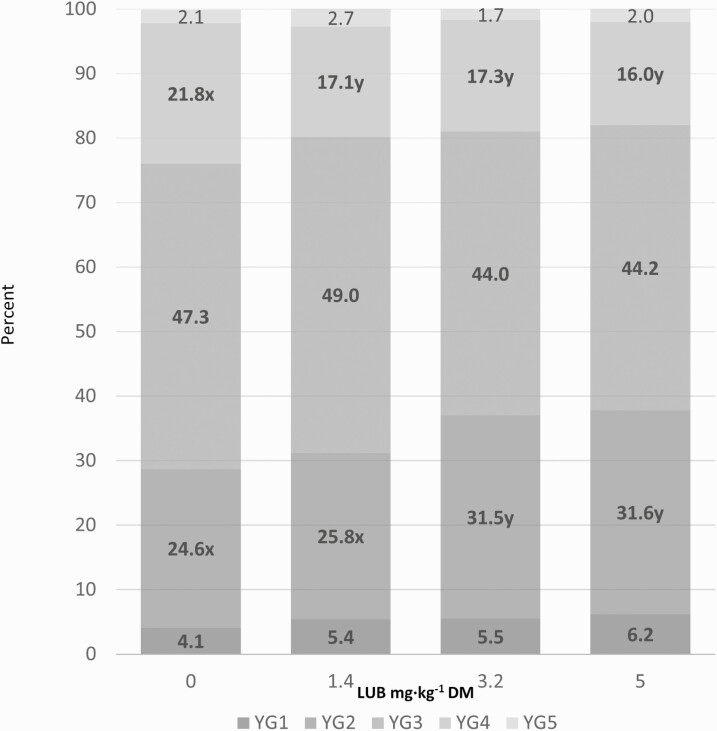
Distribution of yield grades. Discrete yield grade (YG) expressed as a proportion of the cattle in a particular yield grade category to the number cattle graded within each treatment. Within a yield grade category, means from a dose with a different letter differed (*P*_dose_ ≤ 0.05) from each other. Values represented in this figure are arithmetic means, whereas the denoted differences are between the least squares means calculated using PROC GLIMMIX and represent the probability of cattle in a pen displaying a given response.

**Figure 2.**
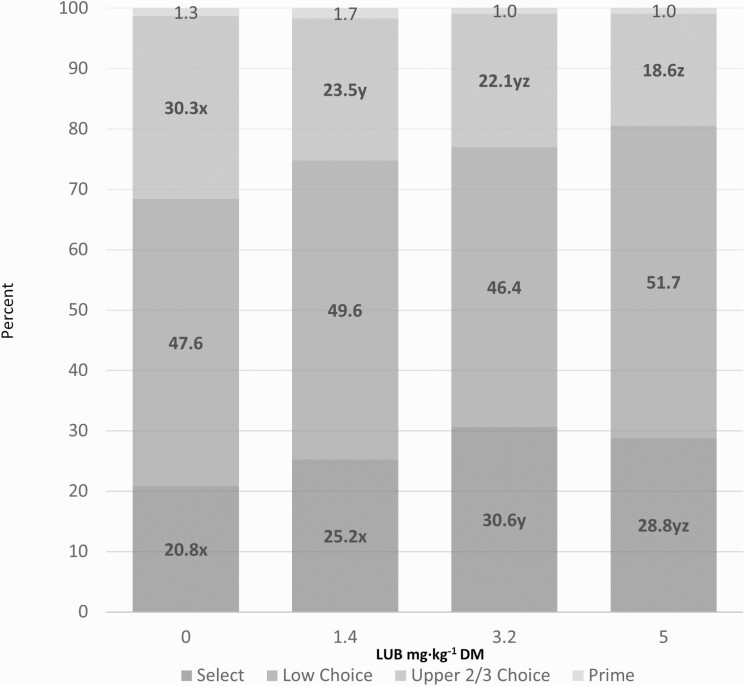
Distribution of quality grades. Quality grade expressed as a proportion of a particular quality grade category to the cattle graded within each treatment. Within a quality grade category, means from a dose with a different letter differed (*P*_dose_ ≤ 0.05) from each other. Values represented in this figure are arithmetic means, whereas least squares means calculated using PROC GLIMMIX and representing the probability of cattle in a pen displaying a given response.

Dressing percentage increased 0.7 to 1.2 percentage points and ribeye area increased 3.3 to 5.7 cm^2^ for LUB-treated cattle compared with those in the CON group (*P*_trt_ < 0.001). Marbling score decreased 13 to 27 units (*P*_trt_ < 0.001) and yield grade decreased 7 to 20 points (*P*_trt_ < 0.001) compared to CON. The frequency of excessively trimmed carcasses was 1.1% (8/707), 1.4% (10/702), 1.8% (13/708), and 1.4% (10/707) for the CON, 1.5, 3.5, or 5.5 mg·kg^-1^ LUB groups, respectively (data not shown). The frequency of dark cutter carcasses was 0.3% (2/707), 0.6% (4/702), 0.3% (2/708), and 0.1% (1/707) for the CON, 1.5, 3.5, or 5.5 mg·kg^−1^ LUB groups, respectively (data not shown). The distribution of yield grades and quality grades are shown in Figures 1 and 2, respectively. Yield grade 2 increased (*P*_trt_ ≤ 0.05) in the 3.5 and 5.5 mg·kg^−1^ LUB groups and yield grade 4 decreased (*P*_trt_ ≤ 0.05) for all LUB groups compared to CON. The proportion of carcasses grading Select increased (*P*_trt_ ≤ 0.05) in the 3.5 and 5.5 mg·kg^−1^ LUB groups and the proportion of carcasses grading Upper 2/3 Choice decreased (*P*_trt_ ≤ 0.05) for all LUB groups compared to CON.

## DISCUSSION

LUB is the first FDA-approved drug that reduces NH_3_ gas emissions from an animal or its waste. LUB possesses antagonistic behavior at the β _1_ and β _2_ adrenergic receptor subtypes and agonistic behavior at the β _3_ adrenergic receptor subtype in cattle (FDA-FOI, 2018). Two pivotal studies demonstrated the clinical effectiveness of LUB for reducing NH_3_ gas emissions·kg^−1^ BW and HCW when fed to feedlot cattle for 14 to 91 d (FDA-FOI, 2018). The 91-d study demonstrated that feeding LUB at doses as low as 1.5 mg of LUB·kg^−1^ of DM over the last 91 d of the feeding period reduced NH_3_ gas emissions·kg^−1^ BW and HCW. No additional reduction in NH_3_ gas emissions was achieved when LUB was fed at 22 mg·kg^−1^ of DM compared to 5.5 mg·kg^−1^ of DM (FDA-FOI, 2018).

This study was designed to provide data across the approved dose range of 1.5 to 5.5 mg·kg^−1^ DM LUB when fed to cattle for the last 56 d of the finishing period. The 3.5 and 5.5 mg·kg^−1^ DM doses of LUB significantly reduced calculated NH_3_ gas emissions compared to the CON, and all three LUB doses significantly reduced calculated NH_3_ gas emissions compared to CON when standardized by BW and HCW, with reductions increasing as the LUB dose increased.

In a review of feedyard NH_3_ gas emissions, Waldrip et al. (2015) noted that up to 90% of NH_3_ gas emitted from feedyards originates from urine deposited in animal pens. The magnitude of the NH_3_ gas emissions depended on both weather and management practices. They estimated NH_3_ gas emissions for feedyard cattle ranged from 50 to 280 g/d, equivalent to 28% to 72% of fed N, and concluded that managing cattle diets to meet, but not exceed, metabolic CP requirements was likely the most practical way to reduce N losses. They proposed 3 possible mitigation practices: 1) dietary manipulation to decrease N excretion, 2) inhibition of urea hydrolysis, and 3) capture of ionic ammonium in manure with pen-surface amendments (e.g., urease inhibitors, alum, and zeolites). A fourth approach discussed here, involves using a compound, LUB, that is known to decrease NH_3_ gas emissions.

Ammonia gas emissions are difficult to measure accurately in a large pen commercial feedlot. For this study, a cumulative ammonia gas emission equation developed by Brown et al. (2019) from the pivotal study used by FDA to approve LUB and validated by an additional 28 d emission study conducted in steers of varying initial target bodyweight of 272, 363, 454, or 544 kg was utilized to determine calculated ammonia gas emissions. As indicated in the Materials and Methods section, there are five variables in the CCAGE equation. All five variables were highly significant (*P* ≤ 0.0007) for determination of CCAGE (*R*^2^ = 0.9871). Additionally, the range in the values of variables in the emission equation development study encompassed the range in the values observed in the current study (Table 6). This information, taken as a whole, indicates the CCAGE equation is valid to use for this current study and provides a reasonable and accurate estimation of ammonia gas emissions.

**Table 6.** Ranges in values for variables (lubabegron dose, duration of treatment, cumulative nitrogen intake, and ambient temperature) used in CCAGE equation for current study compared to ranges in values from Brown et al. (2019)

Current study												
Cumulative nitrogen intake (kg)												
				LUB (mg·kg^−1^ DM)								
	CON			1.5			3.5			5.5		
Treatment duration (D)	Mean	Min	Max	Mean	Min	Max	Mean	Min	Max	Mean	Min	Max
56	14.2	12.4	15.0	14.5	12.1	15.9	14.4	13.0	15.4	14.4	12.8	15.9
Equation development study (Brown et al. 2019)												
Cumulative nitrogen intake (kg)												
				LUB (mg·kg^−1^ DM)								
	CON			1.4						5.5		
Treatment duration (D)	Mean	Min	Max	Mean	Min	Max				Mean	Min	Max
28	5.9	4.2	7.9	6.2	5.2	7.6				5.9	4.7	7.5
56	11.6	8.6	15.1	12.1	10.5	14.4				11.3	9.4	14.1
91	18.3	14.0	22.5	18.9	17.1	21.1				18.2	15.5	21.1
Current study												
Ambient temperature (°C)												
Treatment duration (D)		Mean		Min						Max		
56		24.9		24.6						25.3		
Equation development study (Brown et al. 2019)												
Ambient temperature (°C)												
Treatment duration (D)		Mean		Min						Max		
28		17.1		3.9						23.4		
56		17.1		3.9						24.9		
91		17.2		3.9						25.7		

Feedlot diets are designed to meet or exceed the complete nutrient requirements for finishing beef cattle (NASEM, 2016). Approximately 10% to 30% of dietary N is utilized by the animal and deposited as protein in tissues, with the remaining N emitted into the environment through fecal and urinary excretion, where it becomes susceptible to volatilization as NH_3_ (Cole et al., 2006; Koenig and Beauchemin, 2013; Waldrip et al., 2015). LUB has been demonstrated to reduce NH_3_ gas emissions. LUB, the active ingredient in Experior, can be included at 1.39 to 5 ppm of complete feed (90% DM basis) to provide 13 to 90 mg LUB per head per day continuously to beef steers and heifers fed in confinement for slaughter as the sole ration during the last 14 to 91 d on feed (FDA-FOI, 2018).

Nitrogen intake is an important determinant of NH_3_ gas emissions (Todd et al., 2013). Because dietary CP levels were the same for all cattle in this study, the differences in DMI reflect differences in N intake. All LUB dosages resulted in higher DMI than CON. Based on the NH_3_ gas emission calculation (Brown et al., 2019), as N intake increases so does NH_3_ gas emissions, yet the animals receiving 3.5 and 5.5 mg·kg^−1^ DM doses of LUB still had significantly lower CCAGE than those in the CON group.

LUB effectively and safely reduces NH_3_ gas emissions per unit of body and carcass weight, which implies improved N utilization by cattle. When fed, LUB is absorbed through the gastrointestinal tract into the bloodstream and distributed to tissues such as muscle and fat where it selectively binds to β-adrenergic receptors. The metabolic activity of LUB in a given tissue depends on the density and subtype (i.e., β _1_, β _2_, or β _3_) of the adrenergic receptor in that tissue. LUB also improves responsiveness to insulin (Appendix O, FDA-EA, 2018) and increases gluconeogenesis (Appendix P, FDA-EA, 2018). The net effect of these changes is more efficient utilization of amino acids and glucose for muscle protein, and decreased circulating amounts of amino acids (Appendix P, FDA-EA, 2018), glucose, and urea-N (Appendix Q, FDA-EA, 2018), which is a waste product of amino acid degradation and the primary precursor of NH_3_ gas emissions. LUB’s mechanism of action at the cellular level leads to phosphorylation of enzymes that triggers a cascade of metabolic events. These ultimately result in a reduction of NH_3_ gas emission (FDA-FOI, 2018), which implies that more N is available for use by the animal, eventually leading to increased synthesis of skeletal muscle protein (Section 6.1; Appendix R, Section 1.3, FDA-EA, 2018).

### Implications for Beef Feedlot Producers

Both the 91-d pivotal study (FDA-FOI, 2018) and the current study demonstrated reductions in NH_3_ gas emissions per unit BW and HCW and increased BW and HCW for steers fed LUB.

Based on the two clinical registration studies submitted to the FDA to obtain regulatory approval of Experior, the range in the reduction in cumulative grams of NH_3_ gas emissions when feeding LUB at either 1.4 or 5.5 mg·kg^−1^ was 7.3 to 9.6% (FDA-FOI, 2018) when fed for 14 d and 8.9% to 11.9% (FDA-FOI, 2018) when fed for 91 d. The range in the reduction of calculated cumulative grams of NH_3_ gas emissions was 1.3% to 11.0% for this study, which is consistent with the NH_3_ gas emissions observed in the clinical registration program.

Since NH_3_ gas emissions are standardized by BW and HCW, the contribution of the animal/carcass weight component to standardized NH_3_ gas emissions reduction can be calculated by determining the percentage of the reduction attributable to the numerator (CCAGE) and the denominator (BW or HCW). Approximately 43.3%, 73.2%, and 85.9% of the reduction in NH_3_ gas emissions standardized by BW is attributable to CCAGE for the 1.5, 3.5, or 5.5 mg·kg^−1^ dose groups, respectively. The CCAGE contribution to the reduction in NH_3_ gas emissions standardized by HCW is slightly lower at 34.2%, 63.2%, and 75.3% for the 1.5, 3.5, or 5.5 mg·kg^−1^ dose groups, respectively, indicating that a higher proportion of the N conserved by a reduction in NH_3_ gas emissions is directed toward the components of the carcass compared to non-carcass components of the animal. These calculations also demonstrate that the reduction in standardized NH_3_ gas emissions is affected positively by changes to the numerator (i.e., decreased CCAGE) and the denominator (i.e., increased BW and HCW), although the majority of the reduction appears to be attributable to a reduction in CCAGE for the 3.5 or 5.5 mg·kg^−1^ dose groups.

Cattle mobility has been an important animal welfare topic for the beef industry (Lyles and Calvo-Lorenzo, 2014), and an industry-wide mobility scoring system (NAMI, 2015) was developed to measure these outcomes. In the current experiment, using this system, the percent of cattle with normal mobility (i.e., mobility score = 1) in lairage prior to slaughter was not different across all treatment groups (82.7%, 80.2%, 81.7%, and 80.9% for CON, 1.5, 3.5, or 5.5 mg·kg^−1^ LUB, respectively). However, percent of cattle with normal mobility was lower than 92.1% of cattle that were scored as normal as published by Edwards-Callaway et al. (2017). Although treatment differences were not seen in abnormal mobility scores (≥2) in the current study, the observation of such scores may be associated with the increased handling and stress of the marketing process. Previous studies evaluating cattle mobility have indicated that other risk factors such as handling intensity, heat stress, transport conditions, lairage conditions, increased slaughter weights, and subclinical laminitis are associated with the multifactorial nature of fed cattle mobility and that the use of growth technologies alone could not confirm a causation relationship with poor mobility (Bernhard et al., 2014; Burson, 2014; Boyd et al., 2015; Thomson et al., 2015; Hagenmaier et al., 2017a, 2017b). The current study is the first to publish health and mortality rates for cattle fed LUB in a large pen commercial setting, and the results demonstrate that there were no significant differences across LUB treatments in abnormal health observations, animals found dead, or animal removals. Overall, these results provide data and information on cattle mobility and health relative to the use of LUB, which are important welfare indicators to report as new technologies come to the market and management strategies are developed to continuously improve cattle welfare in the beef industry.

## CONCLUSIONS

LUB reduced calculated NH_3_ gas emissions·kg^−1^ BW and HCW over the final 56 d of the finishing period. The reduction in standardized NH_3_ gas emissions is affected positively by changes to the numerator (i.e., decreased CCAGE by 85 to 708 g) and the denominator (i.e., increased BW by 11.6 to 14.3 kg and HCW by 11.3 to 17.1 kg), although the majority of the reduction appears to be attributable to a reduction in CCAGE. Feeding LUB did not have negative or adverse effects on animal health or mobility. Additionally, feeding LUB increased ADG (0.19 to 0.26 kg), improved gain efficiency (9.0% to 12.0%), increased dressing percentage (0.7% to 1.2%), increased ribeye area (3.3 to 5.7 cm^2^), and decreased marbling score (13 to 27 units).

LUB is the first product with an environmental claim approved by the CVM. By lowering NH_3_ gas emissions, LUB reduces nitrogen wastage into the environment and, when in excess, nitrogen is detrimental to the environment. Excess nitrogen in the atmosphere can impair the ability to breathe and limit visibility. When excess nitrogen comes back to earth from the atmosphere, it can harm the health of forests, soils, and waterways. LUB is also the first selective β _3_ receptor agonist approved for use in animals, making it a unique and innovative product for cattle producers. LUB is the first pharmaceutical tool with an environmental claim available for use in the cattle industry that enables beef producers the ability to provide beef to consumers in a more environmentally responsible manner. Producers can select the LUB dose and finishing period that aligns to their emissions goals for NH_3_ gas. If producers are faced with more stringent reporting of NH_3_ gas estimates in the future due to NH_3_ role in the formation of PM_2.5_, LUB could be incorporated into their finishing programs to reduce NH_3_ gas emissions. This study showed LUB decreases CCAGE, increases BW, and increases HCW when fed for the last 56 days of the finishing period in feedlot steers.
