# Erratum to: Effects of various doses of lubabegron on calculated ammonia gas emissions, growth performance, and carcass characteristics of beef cattle during the last 56 days of the feeding period

**DOI:** 10.1093/tas/txab210

**Published:** 2021-11-15

**Authors:** John C Kube, Ben P Holland, Alyssa B Word, Janet B Allen, Michelle Calvo-Lorenzo, David McKenna, Gary Vogel

**Affiliations:** 1 Elanco, Greenfield, IN 46140, USA; 2 Cactus Research, Amarillo, TX 79116, USA; 3 Tyson Fresh Meats Inc., Dakota Dunes, SD 57049, USA

In “Effects of various doses of lubabegron on calculated ammonia gas emissions, growth performance, and carcass characteristics of beef cattle during the last 56 days of the feeding period” by Kube et al. [https://doi.org/10.1093/tas/txab137], there were several errors, which are listed in this erratum as follows:

In the “Ammonia Gas Emissions Calculations” section, the equations following (Brown et al., 2019) have been corrected.

In Table 4, the “Difference from control” columns were incorrectly formatted. These have been corrected online.

